# Case Report: An Extremely Rare Case of Epitheloid Type Leiomyoma

**DOI:** 10.1155/2024/2259872

**Published:** 2024-09-27

**Authors:** Mokhamad Zhaffal, Shazia Tariq, Anastasia Salame

**Affiliations:** ^1^ Obstetrics and Gynecology Department Kanad Hospital, Al Ain, UAE; ^2^ Reproductive Endocrinology and Infertility Fakih Medical Center, Al Ain, UAE

**Keywords:** epithelioid, epithelioid smooth muscle tumor, leiomyoma, leiomyomas, plexiform tumorlet, uterine myomectomy

## Abstract

**Introduction:** Uterine leiomyomas are a frequent finding in women of reproductive age. However, rare, unusual growth patterns exist, such as atypical, cellular, mitotically active, myxoid, and epithelioid leiomyomas, and present a major concern as they mimic highly malignant uterine tumors such as uterine leiomyosarcomas. An example of such cases is the epithelial type leiomyoma which is the subject of our report in a 35-year-old female.

**Case:** A 35-year-old, nulliparous lady presented with abnormal uterine bleeding to the emergency department. Workup revealed a suspicious uterine mass that was extending from the intramural part of the fundus down the cervical os. The patient was managed by open myomectomy without complications. The final pathology report revealed an extremely rare benign epithelial type leiomyoma, no malignancy, areas with minimal atypia and low mitotic activity (< 5/10 high power fields), and areas of focal necroses (possibly ischemic type) were seen. No coagulative tumor necrosis was noted.

**Conclusion:** Uterine leiomyomas are a common pathology of the uterus that can be confused with malignant tumors, especially in the setting of unusual growth patterns such as the epitheloid leiomyomas. Definitive management involves myomectomies with regular follow-up with favourable prognosis. It is important to properly manage these cases to avoid the overdiagnosis and mistreatment not to mention the repercussion of false diagnoses on the patient's mental health and well-being.

## 1. Introduction

Fibroids, known as myomas or leiomyomas, are the most common benign smooth muscle tumors (SMT) affecting the female reproductive system. Up to 70% of women in the reproductive age group are diagnosed with pelvic fibroids [1]. Despite being benign in nature and frequently asymptomatic, uterine fibroids can present with cumbersome symptoms such as abnormal uterine bleeding (AUB), pelvic heaviness, pain, constipation, increased abdominal girth, infertility, miscarriages, and preterm labor [1–3]. Uterine fibroid pathogenesis is multifactorial and predisposing factors can be divided into age-related, race-related, genetic factors, and exposure factors.

The most common type of fibroids encountered is the spindled leiomyoma; however, some unusual growth patterns have been documented in the literature. These include atypical, cellular, mitotically active, myxoid, and epithelioid leiomyomas [4]. The latter is an extremely rare tumor of the uterus that frequently is misdiagnosed as a leiomyosarcoma based on its behaviour and appearance on imaging [5].

We present a case report of uterine fibroid with unusual appearance on imaging and inconclusive diagnosis of the pathology of the targeted biopsies taken during a diagnostic hysteroscopy. The final surgical pathology received after the open myomectomy revealed a rare epitheloid-type leiomyoma.

## 2. Case Presentation

A 35-year-old lady, G0P0, not sexually active, presented with an acute episode of AUB to the emergency department (ED). The patient had no relevant medical history. The patient's surgical history was significant for an open myomectomy done in 2014 and a hysteroscopic endometrial biopsy done in 2020 for AUB as well. No operative or histopathology reports were available for both procedures, but as per the patient, the pathology report was within normal. The patient had no significant family history.

**Figure 1:**
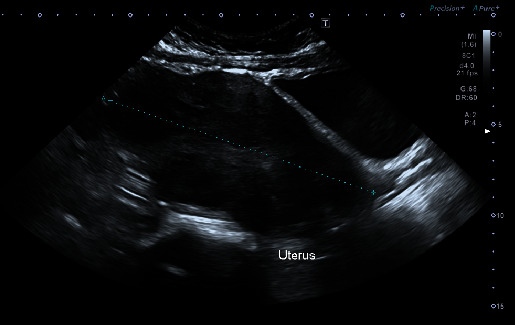
Transabdominal ultrasound of the patient: multiple intramural fibroids, thick poorly defined endometrium.

Upon presentation, the patient noted having dizziness and fatigue upon exertion. Vital signs were stable. Blood tests ordered in the emergency department revealed a hemoglobin of 5.7 g/dL. The remainder of the blood test results ordered are presented in Table 1. Transabdominal scan done (as the patient was not sexually active) showed an anteverted bulky uterus measuring 15.3 × 8.9 × 7.7 cm. Multiple intramural fibroids were noted the largest of which measured 46 × 35 mm in the fundal region. A thick poorly defined endometrium was noted, however, no further comment could be done on the status of the endometrium given that the scan was performed transabdominally (Figure 1).

**Figure 2:**
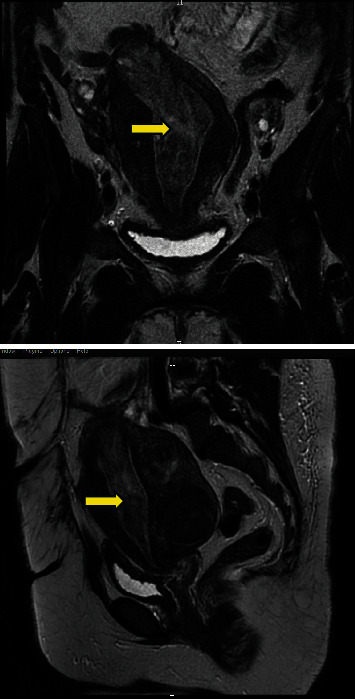
Pelvic MRI with gadolinium revealed an anteverted and bulky due to large fibroids within the posterior wall. Two intramural (FIGO 4) fibroids measuring 4.6 × 4.0 and 4.5 × 3.0 cm, respectively, were noted. Endometrium appeared thickened with a suspicion of a large pedunculated mass filling the whole uterine cavity, extending from the fundus to the internal cervical os.

In the ED, the patient received medical management in the form of tranexamic acid IV and progestin pills (norethisterone 10 mg once orally) in addition to the transfusion of two units of packed red blood cells. Repeat HGB increased to 8.5 g/dl and bleeding stopped after treatment. She was discharged on oral tranexamic acid, oral iron, and norethisterone 10 mg once daily for 10 days to perform a pelvic MRI as an outpatient later. Pelvic MRI with gadolinium revealed an anteverted and bulky due to large fibroids within the posterior wall. Two intramural (FIGO 4) fibroids measuring 4.6 × 4.0 and 4.5 × 3.0 cm, respectively, were noted. Endometrium appeared thickened with a suspicion of a large pedunculated mass filling the whole uterine cavity extending from the fundus to the internal cervical os (Figure 2).

Based on the MRI report, the patient underwent a diagnostic hysteroscopy for better evaluation of the endometrial cavity mass. Diagnostic hysteroscopy revealed a lower uterine segment polypoid endometrium with a large irregular mass originating from the fundus around 7 cm in size with hypervascularity and areas of possible necrosis. Targeted biopsies were taken from the mass and sent to histopathology. The pathology report came back inconclusive on the nature of the mass with a comment of inactive type endometrium and myometrium with hemosiderin depositions without evidence of hyperplasia, atypia, or malignancy.

An open myomectomy was performed after extensively counselling the patient regarding the surgical approaches in view of her age, marital status, nulliparity, risk of malignancy, and indefinite diagnosis of the targeted biopsy taken.

During the procedure, four intramural myomas were removed in addition to an elongated mushroom-like structure with areas of necrosis measuring around 7–8 cm (submucous myoma that was originally biopsied during the hysteroscopy). The estimated blood loss was 300 mL, and the patient's postoperative course was uneventful. The patient was discharged home on Day 4 postoperatively to follow up in the clinic on a regular basis.

**Figure 3:**
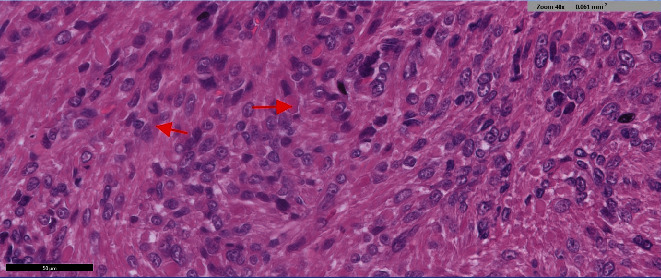
Final pathology: epitheloid cells (see arrows); areas with minimal atypia and low mitotic activity (< 5/10 high power fields) were noted.

The final pathology showed no malignancy with the mass being an epithelioid leiomyoma. Areas with minimal atypia and low mitotic activity (< 5/10 high power fields) were noted. Areas of focal necroses (possibly ischemic type) were seen but no coagulative tumor necrosis (Figure 3).

Written consent was obtained from the patient with permission to use the radiology images and pathology slides.

## 3. Discussion

Uterine fibroids or leiomyomas are a common finding in reproductive age group women. Usually, the diagnosis of such tumors is straightforward, and management is based on the symptomatology of the patient. However, in the setting of the unusual growth pattern leiomyomas, confusion might arise given the atypical presentation both on the imaging as well as during the surgical treatment. Those tumors are frequently mistaken for leiomyosarcomas, with the confusion to be resolved only after the final pathology report is out [5].

Of the proposed treatments for such tumors is myomectomy. Given the suspicious appearance of those tumors as well as the fear of dissemination of undiagnosed leiomyosarcomas, the open myomectomy approach is favoured to avoid morcellation in the laparoscopic approach [6]. As per a case review published in 2013, myomectomy was found to be a safe and reassuring treatment approach, especially in patients who are interested in preserving their fertility. However, caution should always be noted about any residual disease, as well as the possibility of recurrence of the disease in the future [7]. Thus, regular follow-up of those patients is recommended. This is particularly important in the setting of at least 2 of the following findings: tumors measuring more than 6 cm, 2–4 mitotic figures/10 hpf, and moderate to severe cytological atypia and necrosis [8].

In our patient, the tumor size was 7 cm, with minimal atypia and areas of necrosis. The mitotic score reported was less than 5 mitotic figures. The tumor was defined as benign but the patient was scheduled for regular follow-up in the outpatient clinic to monitor for recurrence given her history of open myomectomy and previous episodes of AUB that were suboptimally diagnosed. Based on the patient's history and due to the lack of the previous surgical and pathology reports, it is difficult to say whether the previous presentations were due to a similar pathology with recurrence of the epitheloid leiomyoma that was presently diagnosed.

## 4. Conclusion

Uterine leiomyomas are a common pathology of the uterus that can be confused with malignant tumors, especially in the setting of unusual growth patterns such as the epitheloid leiomyomas. Definitive management involves myomectomies with regular follow-up with favourable prognosis.

## Figures and Tables

**Table 1:** Blood test results of the patient done in the emergency room.

**Test**	**Result**
Hemoglobin	5.7 g/dL
Platelets	435 k
TSH	2.02 uIU/mL
Beta hCG	negative
Prolactin	7.03 ng/mL
FSH	4.99 mIU/mL
Estradiol	67.68 pg/mL

## Data Availability

The data that support the findings of this study are available on request from the corresponding author. The data are not publicly available due to privacy or ethical restrictions.
